# Nerve Growth Factor Compromise in Down Syndrome

**DOI:** 10.3389/fnagi.2021.719507

**Published:** 2021-08-09

**Authors:** Sonia Do Carmo, Benjamin Kannel, A. Claudio Cuello

**Affiliations:** ^1^Department of Pharmacology and Therapeutics, McGill University, Montreal, QC, Canada; ^2^Department of Neurology and Neurosurgery, McGill University, Montreal, QC, Canada; ^3^Department of Anatomy and Cell Biology, McGill University, Montreal, QC, Canada; ^4^Department of Pharmacology, Oxford University, Oxford, United Kingdom

**Keywords:** Alzheimer’s, Down syndrome, nerve growth factor, metabolic pathway, basal forebrain cholinergic neuron, cholinergic dysfunction, neuroinflammation

## Abstract

The basal forebrain cholinergic system relies on trophic support by nerve growth factor (NGF) to maintain its phenotype and function. In Alzheimer’s disease (AD), basal forebrain cholinergic neurons (BFCNs) undergo progressive atrophy, suggesting a deficit in NGF trophic support. Within the central nervous system, NGF maturation and degradation are tightly regulated by an activity-dependent metabolic cascade. Here, we present a brief overview of the characteristics of Alzheimer’s pathology in Down syndrome (DS) with an emphasis on this NGF metabolic pathway’s disruption during the evolving Alzheimer’s pathology. Such NGF dysmetabolism is well-established in Alzheimer’s brains with advanced pathology and has been observed in mild cognitive impairment (MCI) and non-demented individuals with elevated brain amyloid levels. As individuals with DS inexorably develop AD, we then review findings that support the existence of a similar NGF dysmetabolism in DS coinciding with atrophy of the basal forebrain cholinergic system. Lastly, we discuss the potential of NGF-related biomarkers as indicators of an evolving Alzheimer’s pathology in DS.

## Introduction

Down syndrome (DS), also known as trisomy 21, is a genetic disorder caused primarily by the triplication of chromosome 21, which leads to several abnormalities and lifelong intellectual disability. As DS individuals age, they become at a very high risk of developing Alzheimer’s disease (AD). Indeed, DS is now recognized as the most common form of genetic AD, and AD presentation in DS (DSAD) is similar to that of autosomal-dominant AD (ADAD) (Lott and Lai, 1982; Zigman and Lott, 2007; Davidson et al., 2018; Strydom et al., 2018). Therefore, individuals with DS will inevitably develop full-blown AD pathology with extracellular amyloid plaques, intracellular neurofibrillary tangles, neuroinflammation, cholinergic depletion and cognitive and learning deficits leading to clinical dementia in 70% of DS people over 60 years of age (McCarron et al., 2014).

## Alzheimer Pathology in DS

### Amyloid and Tau Pathologies

Due in part to the triplication of genes encoding amyloid precursor protein (APP) and β-amyloid cleavage enzyme 2 (BACE2) (St George-Hyslop et al., 1987; Acquati et al., 2000), located on chromosome 21, individuals with DS display a progressive accumulation of amyloid-beta (Aβ) peptides starting before birth (Lemere et al., 1996; Teller et al., 1996; Mori et al., 2002; Iulita et al., 2014). As in ADAD, the AD pathology in DS (DSAD) follows a predictable disease trajectory (Wiseman et al., 2015; Carmona-Iragui et al., 2017). As early as childhood, a fraction of people with DS present diffuse Aβ plaques within their brain (Lemere et al., 1996; Leverenz and Raskind, 1998). Early Tau pathology (detected as AT8 immunoreactivity) in DS appears by middle age (30–40 years) (Head et al., 2003; Davidson et al., 2018), after Aβ pathology is established, and follows a distribution pattern resembling that of AD, starting in the entorhinal cortex and spreading to the hippocampus and the neocortex (Davidson et al., 2018). By 40 years old nearly all DS brains show advanced AD pathology with extensive amyloid plaques and neurofibrillary tangles (NFTs) (Mann, 1988; Lemere et al., 1996; Leverenz and Raskind, 1998; Lott and Head, 2001; Mori et al., 2002; Head et al., 2003). However, DS brains with AD pathology present a higher density of NFTs than that seen in sporadic AD (Hof et al., 1995). A contributing factor may be the triplication of the dual-specificity tyrosine phosphorylated and regulated kinase 1A gene (DYRK1A), also located on chromosome 21, which is known to phosphorylate Tau at several sites relevant to AD (Woods et al., 2001; Liu et al., 2008). The triplication of APP, PS1 and several immune response mediators associated with AD may also play a role (Arron et al., 2006; Ryoo et al., 2008; Ryu et al., 2010; Kurabayashi et al., 2015; García-Cerro et al., 2017). As in sporadic AD, Aβ seems to be the main driver of dementia in DS as indicated by case studies reporting on individuals with DS who had partial trisomy 21 but were disomic for APP and who did not develop plaques, NFTs or dementia (Prasher et al., 1998; Doran et al., 2017). However, as in AD, cognitive decline in DS shows a stronger association with NFTs than with Aβ plaques (Margallo-Lana et al., 2007). Recently, a comprehensive revision of the order and changes in AD biomarkers in adults with DS has been communicated by Fortea and collaborators (Fortea et al., 2020).

It is noteworthy that the presence of the apolipoprotein E ε4 allele (APOEε4), the highest genetic risk factor associated with AD in the general population, is also a major determinant of AD pathogenesis and progression in people with DS. It has been shown that APOEε4 raises the risk for both early-onset and sporadic AD (Corder et al., 1993; Strittmatter et al., 1993; Qian et al., 2017) and accelerates both symptom onset and pathology severity in a gene-dose-dependent manner (Blacker et al., 1997; Farrer et al., 1997; Fleisher et al., 2013; Liu et al., 2013; Gonneaud et al., 2016; Lautner et al., 2017; Cacciaglia et al., 2018; Mishra et al., 2018). Accordingly, 65–80% of all AD sufferers harbor at least one APOEε4 allele (Farrer et al., 1997). The elevated risk of developing dementia conferred by APOEε4 involves mechanisms associated with both Aβ and tau aggregation (Therriault et al., 2020). APOEε4 carriers also have increased blood-brain barrier breakdown that has been shown to predict cognitive decline (Bell et al., 2012; Zhao et al., 2015; Montagne et al., 2020). Similarly, in people with DS the presence of the APOEε4 allele increases the risk of dementia, although to a lesser extent than in the general population (Prasher et al., 2008; Rohn et al., 2014). It also lowers the age of disease onset (Schupf et al., 1996; Deb et al., 2000; Coppus et al., 2008; Bejanin et al., 2021), aggravates Aβ deposition (Hyman et al., 1995; Bejanin et al., 2021), and accelerates neurodegeneration (Bejanin et al., 2021). Additionally, DS individuals harboring the APOEε4 allele are at additional increased risk for early mortality (Prasher et al., 2008; Hithersay et al., 2019).

### Neuroinflammation

Neuroinflammation is another paramount feature of AD pathology that contributes to the progression and severity of the disease (Akiyama et al., 2000). The interest in the role of immune processes in AD pathogenesis began with the discovery of major histocompatibility molecules and complement system proteins in amyloid plaques (Jonker et al., 1982), and the description of HLA-DR- and IL-1β-positive reactive microglia surrounding amyloid plaques and neurofibrillary tangles (McGeer et al., 1987, 1988). This concept was reinforced by genome-wide association studies indicating that immune-related genes, such as TREM2, HLA-DRB5-HLA-DRB1, CR1 and CLU are risk factors for AD (Harold et al., 2009; Lambert et al., 2009, 2013; Brouwers et al., 2012; Jonsson et al., 2013). DS brains display lifelong neuroinflammatory changes starting at the fetal stage, prior to plaque deposition. Still, the precise cause of neuroinflammation initiation –triggered either by the accumulating AD pathology or by the triplication of immune-related genes [reviewed in Wilcock (2012)]—remains unclear. Early reports on neuroinflammation in DS described a pronounced proliferation of activated glia overexpressing S100B, another chromosome 21 gene product, and interleukin-1 (IL-1) α and β (Griffin et al., 1989; Royston et al., 1999). Since then, the evolving neuroinflammatory phenotype of DS, which presents both similarities and differences compared to that in sporadic AD, has been increasingly described (Stoltzner et al., 2000; Head et al., 2003; Xue and Streit, 2011; Wilcock et al., 2015; Flores-Aguilar et al., 2020). In fetuses and neonates with DS, neuroinflammation is characterized by an increase in the number of IL-1β-expressing microglia (Griffin et al., 1989). This neuroinflammation escalates as children and young adults with DS show an exacerbated neuroinflammatory profile with activation of the complement pathway, elevated levels of key inflammatory cytokines and altered microglia morphology indicative of activation, including the presence of rod-like microglia (Stoltzner et al., 2000; Wilcock et al., 2015; Flores-Aguilar et al., 2020). Older DS individuals (over 40 years of age) also display increased levels of potent inflammatory cytokines compared to karyotypical controls, although to a lesser extent than their younger DS counterparts. However, an increase of dystrophic microglia with age has been reliably demonstrated (Stoltzner et al., 2000; Wilcock et al., 2015; Flores-Aguilar et al., 2020). Accordingly, elevated cytokine expression and immune dysregulation have been reported in the blood of children and adults with DS (Licastro et al., 2005; Iulita et al., 2016; Sullivan et al., 2017; Waugh et al., 2019; Weber et al., 2020). It has been proposed that such changes promote AD pathology in DS (Wilcock and Griffin, 2013). Such changes may also be used to predict and monitor pathological progression. For example, longitudinal changes in TNFα, IL-8, and AD biomarkers in plasma along with a nerve growth factor (NGF) metabolism dysregulation could predict prospective cognitive decline in a population of DS individuals asymptomatic for AD (Iulita and Cuello, 2016).

### Cholinergic Dysfunction

The cholinergic neurotransmitter system is crucial for cortical and hippocampal activity, learning and memory. Its atrophy and degeneration are central to AD symptomatogenesis (Bowen et al., 1976; Davies and Maloney, 1976; Whitehouse et al., 1981, 1982; Mufson et al., 1989; Grothe et al., 2010; Kerbler et al., 2015). Its role in the AD pathology is highlighted by the fact that four of the five drugs currently approved for AD treatment are acetylcholinesterase (AChE) inhibitors, which, by preventing the breakdown of acetylcholine, increase the cholinergic tone resulting in improved cognitive outcomes, as long as sufficient cholinergic terminals persist in the telencephalon (Hampel et al., 2018; Kabir et al., 2019; Marucci et al., 2020). Degeneration of basal forebrain cholinergic neurons (BFCNs) parallels the development of AD pathology, progressing silently for several years prior to the onset of cognitive symptoms (Grothe et al., 2014), as reviewed by Hampel et al., 2018. Further, the degeneration of BFCNs predicts atrophy of the brain regions innervated by their projections such as the entorhinal cortex and cerebral cortex (Schmitz and Spreng, 2016; Schmitz et al., 2018). Loss of cholinergic innervation has also been linked to vascular dysfunction, another early predictor of the progression to AD (Iturria-Medina et al., 2016), and increased blood-brain barrier permeability (Domer et al., 1983; Radu et al., 2017; Nizari et al., 2019, 2021).

Cholinergic dysfunction in DS was first evidenced by a significant reduction in choline acetyltransferase (ChAT) and AChE activity in the temporal cortex of older individuals with DS, which was not present in a younger DS subject (Yates et al., 1980, 1983). Soon after, a significant and seemingly age-related reduction in volume of the nucleus basalis was also observed (Casanova et al., 1985). Further studies demonstrated that abnormalities in the cholinergic system develop as the individuals age and accumulate AD pathology since fetuses display a neuronal density and vesicular acetylcholine transporter (VAChT) immunoreactivity comparable to controls and that newborns with DS have ChAT activity levels similar to age-matched controls (Kish et al., 1989; Lubec et al., 2001). Age-related atrophy and neurodegeneration of BFCNs is recapitulated in mouse models of DS (Holtzman et al., 1992, 1996; Fiedler et al., 1994; Cooper et al., 2001; Granholm et al., 2002) and was attributed to APP gene triplication through disruption of endosomal phenotype and function (Cataldo et al., 2003). Such cholinergic dysfunction is sex-dependent and can be restored by estrogen treatment (Granholm et al., 2002; Kelley et al., 2014b).

Interestingly, in the Ts65Dn mouse model of DS, maternal supplementation with choline, a critical substrate for the synthesis of acetylcholine, during pregnancy and lactation reduced cognitive dysfunction and degeneration of BFCNs in their adult offspring (Moon et al., 2010; Ash et al., 2014; Kelley et al., 2014a; Strupp et al., 2016; Kelley et al., 2016; Powers et al., 2017). Although the exact mechanisms underlying the effects of choline therapy remain obscure, it has been shown that choline treatment rescued the expression of genes related to the cytoskeleton and cholinergic neurotransmission amongst others (Kelley et al., 2019).

## Nerve Growth Factor Metabolic Dysregulation in DS

Basal forebrain cholinergic neurons depend on the continuous supply of NGF for the maintenance of their functional phenotype, their synaptic integrity and ultimately their survival (Hefti and Will, 1987; Cuello, 1996; Levi-Montalcini et al., 1996). In the adult CNS it has been demonstrated experimentally that the levels of *endogenous* NGF regulates the day-to-day number of cortical cholinergic synapses (Debeir et al., 1999). These findings led to Appel’s hypothesis that the trophic support to BFCNs is compromised in AD (Appel, 1981). However, the levels of NGF transcripts are unaffected (Goedert et al., 1986; Jette et al., 1994; Fahnestock et al., 1996) and the protein levels of the NGF precursor, proNGF, are greatly elevated in AD post-mortem brain samples (Fahnestock et al., 1996, 2004; Peng et al., 2004; Pedraza et al., 2005; Al-Shawi et al., 2008; Bruno et al., 2009a). A resolution of such an apparent paradox and insight into the cause of the cholinergic deficits characteristic of AD was brought about by the discovery of an NGF metabolic pathway controlling the availability of mature NGF (mNGF) as well as its extracellular degradation (Bruno and Cuello, 2006). The pharmacological manipulation of this NGF metabolic pathway has shown it to regulate the cholinergic phenotype of both the cortical synapses and the BFCN cell bodies (Allard et al., 2012, 2018).

**FIGURE 1:**
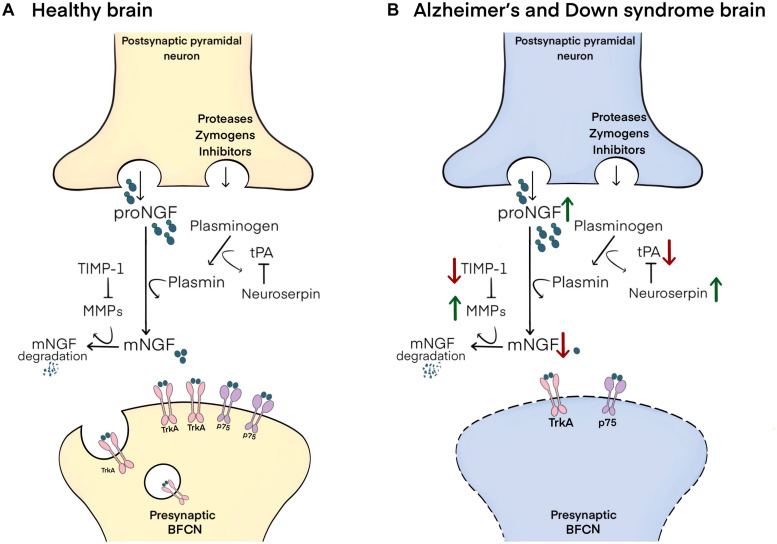
Schematic representation of the NGF metabolic pathway and its altered state in Alzheimer’s and Down syndrome pathology. **(A)** In the healthy brain, proNGF is co-released in the extracellular space with zymogens and convertases involved in its maturation and degradation. proNGF is cleaved to yield mNGF by plasmin, itself derived from the cleavage of plasminogen by tPA, a process regulated by neuroserpin. mNGF then dimerizes and binds to p75/TrkA receptor complexes on presynaptic terminals of BFCNs, followed by the retrograde transport of mNGF to their cell bodies in the basal forebrain. Receptor-unbound mNGF is rapidly degraded by MMP-9 and MMP-3, which are produced from their pro-proteins under the control of TIMP-1. **(B)** In Alzheimer’s disease and in Down syndrome brains, increased neuroserpin and decreased tPA lead to reductions in the maturation of proNGF to mNGF by limiting plasmin concentrations. Further, decreased TIMP-1 and increased MMP-3/MMP-9 result in the excessive degradation of unbound mNGF. These changes result in impaired trophic support to BFCNs, leading to their atrophy.

In brief, proNGF is released into the extracellular space in response to neuronal or neurotransmitter stimulation. In *ex vivo* studies it has been shown that proNGF (and not mature NGF, mNGF) is released along with a set of zymogens and convertases responsible for its maturation and degradation (Bruno and Cuello, 2006). Maturation of proNGF into mNGF is accomplished by the enzyme plasmin, which is generated by the cleavage of its inactive zymogen, plasminogen, by tissue plasminogen activator (tPA), a process regulated by the tPA inhibitor, neuroserpin (Bruno and Cuello, 2006). Degradation of receptor-unbound mNGF is performed by the matrix metalloproteinases 9 and 3 (MMP-9 and MMP-3), derived from cleavage of their protein precursors, a process regulated by tissue inhibitor of metalloproteinases-1 (TIMP-1) (Figure 1; Bruno and Cuello, 2006; Pentz et al., 2021b).

Investigations in post-mortem brain tissue, plasma and cerebrospinal fluid (CSF) revealed that NGF metabolic dysfunction is present in the preclinical and clinical continuum of sporadic AD (Peng et al., 2004; Bruno et al., 2009a,b; Mufson et al., 2012; Hanzel et al., 2014; Pentz et al., 2020). Specifically, both NGF maturation and degradation are disrupted at preclinical AD stages as revealed in individuals with no cognitive impairment (NCI) but with high brain β-amyloid (Aβ) levels (HA-NCI). This NGF dysmetabolim correlated with cerebral Aβ and Tau deposition, cognitive performance, and loss of cholinergic synapses (Pentz et al., 2020). NGF dysmetabolism is also found in the brain of people with prodromal AD, also referred to as mild cognitive impairment (MCI), and with clinical AD as represented by increased levels of proNGF, neuroserpin, as well as MMP-3 and MMP-9 activity (Peng et al., 2004; Bruno et al., 2009a,b; Mufson et al., 2012; Pentz et al., 2020). These findings are also in accordance with other accounts of increased proNGF in CSF from people with AD (E Counts et al., 2016), and with the altered expression of MMP-3, neuroserpin, and plasminogen reported in CSF from AD and MCI participants (Hanzel et al., 2014). These findings have also been replicated in transgenic animal models of the AD-like amyloid pathology (Bruno et al., 2009a; Iulita et al., 2017). Further, it was suggested that there is a link between such NGF dysmetabolism and CNS inflammation in the amyloid pathology since injection of Aβ oligomers in the hippocampus of naïve rats provoked both brain inflammation and NGF dysregulation (Bruno et al., 2009a).

Interestingly, a similar NGF dysmetabolism with increased cortical proNGF levels has been reported in DS (Iulita et al., 2014, 2016; Iulita and Cuello, 2016; Caraci et al., 2017), therefore providing an explanation for the cholinergic atrophy in DS (Yates et al., 1983; Kish et al., 1989; Lubec et al., 2001). In DS as in AD, reduced levels of tPA and plasminogen, which are involved in proNGF maturation as well as heightened neuroserpin expression lead to a build-up of proNGF. In parallel, over-activation of MMP-9, the main NGF-degrading protease, leads to increased degradation of the biologically active mNGF protein (Iulita and Cuello, 2014; Iulita et al., 2014). This double hit on the NGF pathway results in decreased availability of mature NGF to sustain trophic support of BFCNs in DS as in AD. Such impairment in NGF metabolism is an early event in DS and is detectable before the clinical presentation of AD. Indeed, increased levels of proNGF, decreased tPA activity and increased MMP-9 activity were detected in conditioned media from primary cultures from fetal DS cortex (Iulita et al., 2014). In addition, levels of proNGF, as well as MMP-1, MMP-3, and MMP-9 activity were found elevated at AD asymptomatic stages in the plasma from a cohort of clinically characterized DS individuals. In this cohort, an elevation of proNGF levels at the 1-year follow-up predicted the extent of cognitive deterioration (Iulita and Cuello, 2016). The association between Aβ and NGF pathway dysfunction was further strengthened by the fact that Aβ load highly correlated with the elevation of proNGF in older DS individuals (Iulita and Cuello, 2016). The presence of an APOEε4 allele in DS individuals, as in other people at risk of AD, may further aggravate the brain’s NGF dysmetabolism. Indeed, APOEε4 mice show upregulated levels of both proMMP9 and MMP9 (Bell et al., 2012).

## NGF Metabolic Pathway Related Biomarkers as Indicators of AD Pathology in DS

The diagnosis of AD in DS is challenging given the underlying DS intellectual disability and the lack of diagnostic criteria and cognitive screening tools adapted to people with DS (Lee et al., 2017). Therefore, validated biomarkers that signal the progression of Alzheimer pathology in DS are presently of great medical importance. Correlations between classical AD biomarkers and cognition are increasingly being established to define the status of this pathology in DS (Fortea et al., 2020). We propose that NGF metabolism-related biomarkers in body fluids should assist in that task.

Analysis of cortical thickness, intracranial volume, fraction anisotropy, and cerebral blood flow employing magnetic resonance imaging (MRI) could identify AD pathology in both DS and sporadic populations (Handen et al., 2020). Alternatively, positron emission tomography (PET) imaging to trace amyloid deposition with compounds such as Pittsburgh Compound B (PiB) and [18F]-florbetaben, commonly used to detect sporadic AD, have shown mixed results in identifying AD within the DS population. It was suggested that since those with DS display a lifelong amyloidoisis that is already very prominent at a young age, amyloid PET may not be of use in tracking the progress of AD (Abrahamson et al., 2019). More recently, a cross-sectional and longitudinal study in individuals with DS showed that it was possible to differentiate MCI-DS from the cognitively stable group using [18F]-AV-45 (florbetapir) PET. Additionally, although PET tracers for Tau have proved a challenge for the field (Robertson et al., 2017), a recent study using the Tau PET tracer [18F]-AV1451 in a small cohort of DS individuals showed that Tau deposition was correlated with age, amyloid deposition, decreased brain volume and reduced glucose metabolism (Rafii et al., 2017). Evaluation of Tau PET tracers using autopsy brain tissue also suggested that the regional distribution of Tau pathology in DS differs from ADAD and sporadic AD (Lemoine et al., 2020). An issue with current neuroimaging studies in DS populations is that the normative atlases being used were developed for the non-DS population, although this is currently being addressed by the creation of atlases for the DS brain (McGlinchey et al., 2020).

The pattern of biofluid biomarker changes in AD in DS have been considered to be largely similar to those in sporadic AD (Rafii et al., 2015). While those with DS have a higher baseline of Aβ peptides due to the triplication of the APP and BACE2 genes located on chromosome 21, an increase in CSF levels of Aβ42 or the Aβ42/Aβ40 ratio relative to this baseline are associated with the onset of AD in DS (Lee et al., 2017). Several studies have demonstrated that changes in plasma Aβ40 and Aβ42 in DS correlate with AD onset (Schupf et al., 2007; Jones et al., 2009; Matsuoka et al., 2009; Schupf et al., 2010; Coppus et al., 2012). As for Tau, increases in CSF total Tau (tTau) and phosphorylated Tau (pTau), have been correlated with AD onset in DS (McGlinchey et al., 2020; Pentz et al., 2021a). Likewise, plasma neurofilament light (NfL), and IL1β, have been shown in multiple studies to reliably distinguish DSAD individuals with DS asymptomatic for AD (aDS) (Petersen and O’Bryant, 2019; Startin et al., 2019; McGlinchey et al., 2020). Of the biomarkers discussed, NfL has emerged as the leading plasma biomarker. With 90% sensitivity and 92% specificity in its ability to distinguish between aDS and prodromal DSAD groups (Petersen and O’Bryant, 2019; McGlinchey et al., 2020). Additional more recently posited biofluid biomarkers include levels of TNF-a, IL-6, IL-10, and S-adenosylhomocysteine (SAH), a change in SAM/SAH ratio and CpG methylation percentage (Lee et al., 2017).

Given that degeneration of the cortical forebrain cholinergic system is a critical factor associated with cognitive decline in AD, both in the general population and in DS, as discussed above, current AD biomarker panels should be enriched by the addition of biomarkers able to monitor cholinergic dysfunction in both research and clinical contexts (Hampel et al., 2018; Cuello et al., 2019; Pentz et al., 2021a). NGF dysmetabolism’s presence within DS and AD brains, and its relationship to cholinergic dysfunction, present the opportunity for the identification of novel biomarkers signifying AD pathology and subtyping for cholinergic dysfunction within DS populations. Analysis of NGF pathway proteins in matched CSF/plasma samples from DSAD and individuals with DS aDS, as well as controls, revealed that the levels of the 50 kDa isoform of proNGF and MMP9 in CSF were competent to identify symptomatic AD from the wider DS population. Both members of the NGF metabolic pathway identified symptomatic AD from the wider DS population with a sensitivity and specificity matching or outperforming that of the classical AD CSF biomarkers pTau, tTau, and the AB42/40 ratio (Pentz et al., 2021a). Importantly, longitudinal increases in 50 kDa proNGF levels in plasma over 1 year correlated to prospective cognitive decline over the subsequent 2 years (Iulita and Cuello, 2016), demonstrating a potential value of NGF-related biomarkers in identifying incipient cognitive decline in this population.

## Conclusion

The nearly inexorable development of the AD pathology and the ensuing dementia in DS individuals is nowadays well-established and has been eloquently summarized by Lott and Head (2019). The growing awareness of this situation has triggered an increased interest and research in unraveling aspects of the AD pathology in DS, as this is the largest population of genetic AD and therefore offers clues regarding the early, preclinical stages of this pathology. A pathology which continues to defy therapeutic intervention.

As discussed in this brief review, the occurrence of NGF dysmetabolism leading to BFCNs dysfunction is now well-established. NGF metabolism-related biomarkers have proven significance in identifying AD pathology at preclinical stages and in monitoring its progression in the AD clinical continuum. This might offer distinctive possibilities of defining differential conditions of cholinergic compromise.

Alzheimer’s disease is presently recognized as being the leading cause of death in DS. Therefore, novel biomarkers signaling the initial, preclinical stages of AD in DS should offer valuable tools for future early therapeutic interventions. A scenario which would spare DS individuals of the onset of clinical AD and which would also provide new therapeutic opportunities for individuals with sporadic AD.

The further investigation of the NGF metabolic compromise in AD should provide clues as to how best re-establish an adequate trophic support for the phenotypic maintenance of BFCNs; the atrophy of which importantly contributes to cognitive decline in AD pathology. If such pharmacological intervention becomes feasible it would halt the progressive atrophy of the BF cholinergic system. An effective pharmacological intervention of a deregulated NGF metabolic pathway would signify restoring mNGF homeostasis at physiological levels and at physiological sites.

## Author Contributions

ACC and SDC designed and outlined the structure and contents of the review. All authors contributed to the writing and revision of the manuscript and approved the submitted version.

## Conflict of Interest

The authors declare that the research was conducted in the absence of any commercial or financial relationships that could be construed as a potential conflict of interest.

## Publisher’s Note

All claims expressed in this article are solely those of the authors and do not necessarily represent those of their affiliated organizations, or those of the publisher, the editors and the reviewers. Any product that may be evaluated in this article, or claim that may be made by its manufacturer, is not guaranteed or endorsed by the publisher.
